# Astrocyte-Derived Neuronal Transdifferentiation as a Therapy for Ischemic Stroke: Advances and Challenges

**DOI:** 10.3390/brainsci12091175

**Published:** 2022-09-01

**Authors:** Siqi Gong, Han Shao, Xiuying Cai, Juehua Zhu

**Affiliations:** Department of Neurology, The First Affiliated Hospital of SooChow University, Suzhou 215006, China

**Keywords:** astrocyte, transdifferentiation, neuron, ischemic stroke

## Abstract

After the onset of ischemic stroke, ischemia–hypoxic cascades cause irreversible neuronal death. Neurons are the fundamental structures of the central nervous system, and mature neurons do not renew or multiply after death. Functional and structural recovery from neurological deficits caused by ischemic attack is a huge task. Hence, there remains a need to replace the lost neurons relying on endogenous neurogenesis or exogenous stem cell-based neuronal differentiation. However, the stem cell source difficulty and the risk of immune rejection of the allogeneic stem cells might hinder the wide clinical application of the above therapy. With the advancement of transdifferentiation induction technology, it has been demonstrated that astrocytes can be converted to neurons through ectopic expression or the knockdown of specific components. The progress and problems of astrocyte transdifferentiation will be discussed in this article.

## 1. Introduction

Following ischemic stroke, neuronal cells in the damaged area die irreversibly, leading to neurological impairments. Brain tissue injury causes neural stem cells in the subventricular zone (SVZ) and subgranular zone (SGZ) to differentiate into neurons, and terminally differentiated glial cells re-enter the cell cycle to undergo proliferation. Neurons and glial cells have a limited capacity for self-repair. After a brain lesion, new neurons created through endogenous neurogenesis account for less than 1% of the overall loss [1]. It is feasible to prevent the necrosis of some neurons in the ischemic penumbra by using treatments such as neural growth factor and neurotrophic factor [2]. The demand for neurons or glial cells after ischemic stroke cannot be fully satisfied by stimulating endogenous neuron and glial cell proliferation. Cell transplantation therapy has been testified to replace the damaged neurons. By transplanting stem/progenitor cells with multi-directional differentiation capacity, such as pluripotent stem cells (PSCs) [3], mesenchymal stem cells (MSCs) [4], and neural stem cells (NSCs) [5], it is possible to differentiate them into neurons in the host brain. However, limited by their ethical, tumorigenic, and inefficient reprogramming issues, researchers have begun to investigate the transformation of somatic cells into neurons. This provides a rich source of neuronal cells through direct transdifferentiation or indirect lineage transformation from other somatic cells such as astrocytes or fibroblasts. With the development of transdifferentiation technology, direct induction of non-neuronal cells such as astrocytes into neuronal cells in vitro and in vivo has become possible. It has been demonstrated that a wide range of human or rodent astrocytes can be transdifferentiated into neurons under certain conditions [6,7]. These encouraging results provide a potential new approach to treating ischemic stroke.

Astrocytes are the most widely distributed type of cells in the mammalian brain. They share primitive progenitor cells with neurons and are closer in their lineage of occurrence. Inhibition of the characteristic signaling of astrocytes and activation of neural phenotypic signaling may promote direct transdifferentiation of astrocytes into neurons without transforming through the stem cell stage. Hence, they are potential candidates for transdifferentiation into functional neurons [8]. When an ischemic stroke occurs, astrocytes are activated and proliferate, resulting in the formation of a glial scar [9]. A glial scar can seal the lesion in the acute phase, preventing the spread of inflammation and damage. On the other hand, glial scars limit the regeneration of axons and thus hinder the recovery of nerve function [10]. If astrocytes can transdifferentiate to replace the lost neurons, it helps in limiting glial scar formation and restricting neural connection after injury. Therefore, increasing attention has been paid to the field of transdifferentiation from astrocytes as a therapy for neuronal replacement after ischemic stroke. Generally, the transdifferentiation methods of astrocytes are divided into direct transdifferentiation and indirect transdifferentiation.

## 2. Transdifferentiation Methods

**Figure 1:**
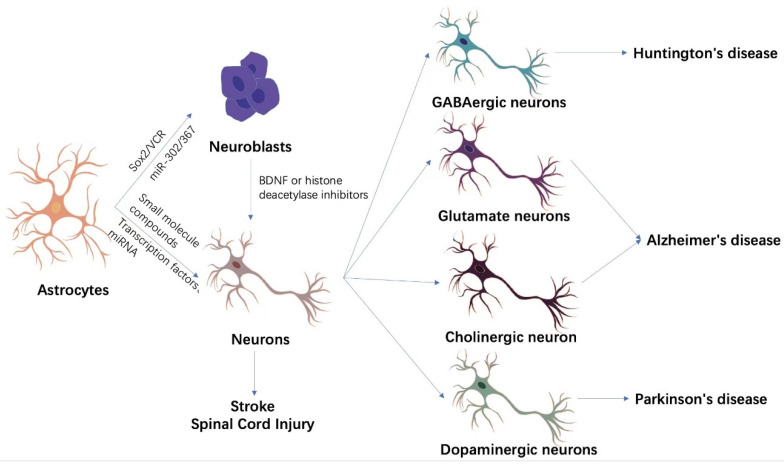
Astrocyte transdifferentiation methods and clinical applications. Astrocyte transdifferentiation into neurons is divided into two main pathways, indirect lineage conversion and direct transdifferentiation. Indirect lineage conversion mainly applies SOX2, miR-302/367, and VCR to convert astrocytes into neuroblastoma cells with differentiation potential. BDNF or histone deacetylase inhibitors are then used to further promote the differentiation of neuroblastoma cells into neurons. Direct transdifferentiation, on the other hand, directly reprograms astrocytes into neurons without passing through neural stem or neuroblastoma cells by a combination of different transcription factors, miRNAs, or small molecule compounds. The newly generated neurons are able to replenish the neuronal loss due to ischemic stroke or spinal cord injury. Under specific transdifferentiation conditions, astrocytes can directionally differentiate into specific functional neurons, such as GABAergic neurons, glutamatergic neurons, cholinergic neurons, and dopaminergic neurons. These functional neurons are important for some specific diseases such as Huntington’s disease, Alzheimer’s disease, Parkinson’s disease, etc.

It has been demonstrated that astrocytes can be reprogrammed to neuronal cells without introducing other regulatory factors after ischemic stroke induced by middle cerebral artery occlusion (MCAO) [11]. Through labeling astrocytes of MCAO rats with plasmids expressing GFP, it was found that some astrocytes expressed the neuronal marker NeuN at about 2–4 weeks after ischemic stroke onset, and over 90% of the transformed neurons expressed dopamine or glutamate receptors on their membranes [12]. However, it has been reported that the transdifferentiation efficiency was very low, and in vitro experiments showed that the efficiency was about 0.15–0.3% of labeled astrocytes [11]. However, another study demonstrated that the cultured astrocyte-derived neurospheres mainly differentiated into astrocytes and oligodendrocytes rather than neurons [13] when transplanted into neonatal or adult mouse brains. This limits its ability to induce new neurons after ischemic stroke, so additional genetic or epigenetic modifications are required for neuronal replacement (Figure 1).

### 2.1. Indirect Lineage Conversion

Indirect lineage conversion of astrocytes refers to the induction of somatic cells to an intermediate transition state by specific transcription factors that regain differentiation potential, and then they differentiate into another somatic cell type. It takes about 7 weeks to produce neurons in vivo [14].

#### 2.1.1. Transcription Factors

SOX2 is a common transcription factor in the indirect spectrum transformation of astrocytes. It has been found that astrocytes in the mouse striatum can be induced to form Achaete-Scute Family BHLH Transcription Factor 1 (Ascl1)-positive intermediate progenitor cells by viral-mediated SRY-Box Transcription Factor 2 (Sox2). These were later proliferated and gave rise to DCX+ neuroblasts [15]. These neuroblasts can develop into mature neurons in the presence of brain-derived neurotrophic factors (BDNF). More than 90% of these neurons showed spontaneous synaptic currents, indicating that they formed functional synapses with endogenous striatum neurons and functionally integrated into the local neural networks. When examining Sox2-overexpressing brains within 1 year of virus injection, no histological evidence of tumorigenesis was observed [16]. The feasibility of Sox2-induced indirect lineage conversion in astrocytes was confirmed in the mouse brain and the injured spinal cord of the mouse. Astrocytes isolated from the spinal cord were also transdifferentiated into DCX+ neuroblasts under the overexpression of Sox2 and then formed neurons by induction of BDNF [17].

#### 2.1.2. miRNA

miRNA is a class of non-coding single-stranded RNA molecules involved in the post-transcriptional regulation of gene expression. Some miRNAs are specifically highly expressed in the central nervous system. Therefore, researchers began to try to transdifferentiate fibroblasts and oligodendrocytes [18] into neurons by miRNAs and obtained some results. It was demonstrated that adult human/mouse astrocytes can be reprogrammed into neuroblasts by miR-302/367 in vivo and in vitro [19,20]. The differentiation of neuroblasts into neurons can be promoted by adding valproic acid (VPA). About 80% of the cells expressed the excitatory neuronal phenotype by examining marker expression in differentiated neurons 6 weeks after transfection. And at the 2-month follow-up, no teratoma formation was observed [19].

#### 2.1.3. Small Molecule Compounds

In addition to converting astrocytes into neuroblasts via transcription factors and miRNAs, researchers found that a combination of “VCR” small molecules composed of Valproic acid, ChIR99021, and Repsox could transform mouse astrocytes into neuroblasts [21]. However, when this finding was applied to human astrocytes, it did not induce noticeable neuron-like morphological changes in cultured human astrocytes [21]. Therefore, if it is to be applied to human astrocytes, other small molecule compounds may also need to be introduced.

### 2.2. Direct Transdifferentiation

Direct transdifferentiation of astrocytes can be induced by transcription factors, miRNA, or small molecule compounds to transdifferentiate astrocytes into neurons. This transdifferentiation can occur both in vivo and in vitro and can reduce tumorigenicity and accelerate the efficiency of transformation [22]. It has been shown that direct transdifferentiation of astrocytes to neurons takes about 2–3 weeks [12], while indirect transdifferentiation takes about 7 weeks [14].

#### 2.2.1. Transcription Factors

Several transcription factors have been shown to induce direct transdifferentiation of astrocytes into neurons through in vitro experiments. In 2002, laboratory studies showed that astrocytes that mediate Paired Box 6 (Pax6) overexpression in vitro can produce neurons [7]. In a stab wound lesion model, Pax6-induced astrocytes produced some cells expressing early neuronal markers [23]. However, these studies did not testify whether the induced cells exhibited neuronal function and their specific neuronal phenotype. Therefore, in 2007, a study reprogrammed postnatal mouse cerebral cortical astrocytes through Neurogenin-2 (Ngn2) and Mammalian Achaete-Schute Homolog 1 (Mash1)/Pax6. Over 85% of transdifferentiated cells produced progeny with neuronal properties. Neurons derived from astrocytes matured more slowly than neurons derived from embryos [24]. Early in culture, co-cultured cortical neurons already had substantial synaptic input, but astrocyte transdifferentiation produced neurons without synaptogenesis [24]. There has been evidence that different transcription factors may regulate stem cells to generate specific neuronal populations [25]. In astrocyte transdifferentiation, it has also been found that neurotransmitter fate selection of nascent neurons can be controlled by selective expression of different neurogenic transcription factors. Forced expression of Ngn2 [26] or Neuronal Differentiation 1 (NeuroD1) [27] induced cortical astrocytes to generate glutamatergic neurons in vitro, whereas forced expression of Distal-less Homeobox 2 (Dlx2) [26] or Ascl1 [28] induced astrocytes to generate GABAergic neurons.

Transcription factors not only induce the conversion of astrocytes into neurons in vitro but also play an influential mediating role in vivo. WhenNeuroD1 was introduced into the damaged area/intact intracranial astrocytes, about 90% of the host astrocytes were transdifferentiated into glutaminergic neurons [27]. The transformed neurons were able to connect with surrounding neural networks, thereby improving motor and cognitive functions in mice [29,30,31]. NeuroD1-mediated astrocytes were effective in producing new neurons 10–30 days after ischemic stroke [32]. In addition, the introduction of transcription factors Ascl1, POU Class 3 Homeobox 2 (Brn2), and Myelin transcription factor 1-like (Myt1l) into human astrocytes can also make them transdifferentiated into neurons and integrated into the neural network. However, its transdifferentiation efficiency was very low, ranging from 0.4% to 5.9% [33]. Later, researchers found that overexpression of Brn2 alone also induced the transformation of mouse astrocytes into functional neurons [34]. To demonstrate the effect of the environment on direct neuronal reprogramming in the adult brain in vivo, the introduction of Ngn2 into astrocytes induced new neurons from the adult neocortex and striatum. These two regions responded differently to Ngn2, with GABAergic neurons predominantly induced in the striatum and glutamatergic neurons primarily generated in the neocortex [35].

The introduction of most experimental transcription factors requires viruses as vectors. This transdifferentiation pathway may have the risk of transgene reactivation, insertional mutagenesis, and residual expression [36]. Therefore, some researchers ameliorated this potential risk through non-viral nanoparticle mediation. PBAE 536 was selected as the vector to introduce the Sox2 gene into primary human astrocytes with a transformation efficiency of about 45%, with good gene expression efficiency, and low cytotoxicity [37].

#### 2.2.2. miRNA

The combination of miRNAs with transcription factors or small molecule compounds has been shown to direct the transdifferentiation of astrocytes into functional neurons. The transcription factors NeuroD1, Ascl1, LIM Homeobox Transcription Factor 1 Alpha (Lmx1a), and miR-218 has been used to induce the transdifferentiation of astrocytes into dopaminergic neurons [38]. Later, it was found that the induction combination of miR-124 combined with the small molecule compounds ruxolitinib, SB203580, and Forskolin can promote the transdifferentiation of astrocytes to neurons. Identification of neuronal subtypes was performed on day 7 of induction, with 25.9% CHAT+ cholinergic neurons and 22.3% VGLUT1+ glutamatergic neurons [39]. Because of the short half-life in vivo, miRNA is usually degraded within a few days. Therefore, the use of miRNAs is laborious and costly.

#### 2.2.3. Small Molecule Compounds

Due to the low cost, ease of synthesis, high reprogramming efficiency, and safety considerations, small compounds have been testified to transdifferentiate into specific neurons in recent years. It was previously mentioned that the “VCR” small molecule combination could not induce significant neuron-like morphological changes in cultured human astrocytes [21]. The researchers added ISX-9, i-Bet151, and Forskolin to various “VCR” small molecules to induce direct transdifferentiation of adult astrocytes into neurons. This transformation method turned out to be viable and functional after transplantation into mice [40]. Another study successfully persuaded the transdifferentiation of fetal brain astrocytes into neurons using a combination of MCM (LDN193189, SB431542, TTNPB, Tzv, CHIR99021, valproic acid, DAPT, SAG, and Purmo). These newborn neurons were transplanted into the brain of mice and could survive for more than 1 month and integrated into the mouse neural circuit. However, the induction of spinal astrocyte transdifferentiation with the MCM combination caused massive cell death [41]. It may be because different glial cell lines were sensitive to different groups of small molecules.

To simplify the combination of small molecule compounds, a recent study applied three each of DAPT, SB431542, LDN193189, and CHIR99021 or their respective functional analogs to transdifferentiate human fetal astrocytes into neurons. It also found that astrocytes in the cortex differentiated mainly into glutamatergic neurons. At the same time, astrocytes in the midbrain differentiated not only into glutamatergic neurons but also partially into GABAergic neurons. This may be due to the fact that different lineages of astrocytes originating from other brain regions could be chemically transformed into different subtypes of neurons [42]. In addition, the “FICB” combination of Forskolin, ISX-9, CHIR99021, and I-BET151 was shown to induce the conversion of mouse astrocytes into neurons in vivo. Later, DBcAMP and Y-27632 were added to the “FICB” combination to optimize the combination to form the “DFICBY” combination. This combination successfully induced astrocytes in the mouse striatum to GABAergic neurons and astrocytes in the cortex to glutamatergic neurons [43]. Small molecule compounds are challenging to apply in clinical treatment because of the diversity of their combinations and the uncertainty of the time and sequence of their addition, which can lead to difficulties in controlling the properties and efficiency of transdifferentiation to generate neurons.

## 3. Transdifferentiation Efficiency

The conversion efficiency of transdifferentiation varies significantly between different methods (Table 1). The VCR cocktail combination induced the generation of DCX+ cells >30% on day [10] and NeuN+ cells >20% on day [16] of infection. Using only VPA and CHIR99021 on transformation efficiency was not significant, but neither CHIR99021 nor Repsox alone nor their combination induced the formation of neuroblasts [21].

Direct transdifferentiation is generally more efficient than indirect transdifferentiation. Among transcription factors, the overall efficiency of Dlx2-mediated neuronal reprogramming was lower than that of Ngn2. The transdifferentiation efficiency of Ngn2 after 10 days of induction was >70%, while Dlx2 was >35.9%. In addition, Ascl1-induced conversion of cortical astrocytes to neurons was only half as efficient as Ngn2 [26]. Ascl1 transformed cortical astrocytes into neurons, of which 13.2 ± 4.2% were glutamate neurons, and 6.5 ± 2.2% were GABAergic neurons [28]. The transdifferentiation efficiency of forced expression of Ngn2 to induce glutamatergic neurons from astrocytes was about 70%, and the transdifferentiation rate of introducing Dlx2 to produce GABAergic neurons was about 36% [26]. Transdifferentiation efficiency of NeuroD1 by retroviral introduction into the injury site of mice with traumatic brain injury was over 90% at day 7 [27]. The induction combination of miR-124 combined with the small molecule compounds ruxolitinib, SB203580, and Forskolin had a 41.5% efficiency of transdifferentiation to DCX+ neurons at day 7 of induction. The neuronal subtypes were identified: 25.9% were CHAT+ cholinergic neurons, and 22.3% were VGLUT1+ glutamatergic neurons [39]. Fetal brain astrocytes were induced using a combination of small-molecule compounds MCM, in which 67% of astrocytes were reprogrammed into neurons within 8–10 days, with approximately 88% converted into glutamatergic neurons and 8% into GABAergic neurons [41].

Efficiency varies across transcription factor vectors and different disease conditions. NeuroD1 introduced by retrovirus had >90% transformation efficiency at day 7 [27]. The focal ischemia model introduced into mice by expressing NeuroD1 through the AAV9 system had a conversion efficiency of >70% on day 17, >50% Tbr1+ cortical neurons (containing Cux1+ superficial and Ctip2+ deep cortical neurons) and approximately 10% GABA+ and PV+ inhibitory neurons [29]. About 66% of infected cells were converted to NeuN+ neurons when NeuroD1 was introduced into astrocytes of stroke model mice via lentiviral vector [30]. While the primary rhesus monkey model of focal ischemia was introduced by expressing NeuroD1 through the AAV9 system motor cortex ET-1 induced focal ischemia model, the conversion efficiency at 42 days can be >90% [32].

The efficiency of transdifferentiation is also related to the cellular microenvironment. The transdifferentiation rate of astrocytes was more significant in the cortex than in the hippocampus in the midbrain [34]. The transdifferentiation efficiency at undamaged sites was lower than that at pathological conditions. In addition, using AAV9 as a vector to introduce NeuroD1, the transdifferentiation efficiency of astrocytes in the striatum of intact mice was about 2.42% [31]. In contrast, the transdifferentiation efficiency in stroke mice was >70% at 17 days in vivo. In addition to this, it was been shown that the proliferation rate of activated reactive astrocytes seemed to differ in different pathological conditions, with higher proliferation rates of reactive astrocytes in stab wounds and ischemic strokes [45].

## 4. Transdifferentiation as a Therapy for Ischemic Stroke

The ability of newborn neurons to have intact synaptic function and to integrate into local neural networks is essential for their efficacy in the treatment of ischemic stroke. In certain trials, astrocyte-mediated reprogramming of neurons was incomplete because emerging neurons failed to develop functional presynaptic outputs, posing an apparent barrier to neural network functional restoration. After injecting a current into neurons from reprogrammed astrocytes, no synaptic response was observed in neighboring cortical neurons after the introduction of Ngn2, Mash1, or Pax6 into astrocytes. Postsynaptic maturation took longer in adult neurons than in embryonic neurons [24]. Neuroblasts were shown to grow into electrophysiologically mature neuronal cells that functionally integrated into the local neural network when mice were given histone deacetylase inhibitors [17]. Most studies detected spontaneous and evoked synaptic events, both excitatory and inhibitory, by examining the electrophysiological function of newborn neurons, indicating that these neurons were functionally integrated into local neural networks [15,17,27,28,29,40,41].

Some studies revealed considerable structural [32] and functional repair [29,46] in mice after transplanting newborn neurons into the cranium of an ischemia mouse model. NeuroD1-induced astrocyte introduction into the mouse cranium resulted in a 35% lesser reduction in motor cortex volume over 2 months compared to the control group [29]. By analyzing the forelimb fine movements in mice, NeuroD1 treatment was found to be beneficial in the recovery of motor deficits after ischemic stroke [46]. NeuroD1 was introduced into the rat amygdala, and auditory fear regulation showed that NeuroD1 was able to rescue the defect of fear memory [29]. The researchers observed no tumor growth in mice treated with SOX2-expressing lentivirus for transdifferentiated tumorigenicity [15]. Teratoma formation in the striatal area of mice treated with viral particles expressing miR-302/367 and VPA 2 months later was likewise not found [35].

## 5. Applications in Other Diseases

In addition to ischemic stroke, many researchers have been exploring the prospect of glial cell transdifferentiation in other diseases. Overexpression of NeuroD1 and Dlx2 induced direct transdifferentiation of astrocytes in the striatum into GABAergic medium spiny neurons (MSNs). It was confirmed that newborn MSNs improved striatum atrophy and motor function in mouse models of Huntington’s disease [47]. Regarding applying astrocyte transdifferentiation in Parkinson’s disease, one group used NeuroD1, Ascl1, Lmx1a, and miR-218 to convert astrocytes into dopaminergic neurons in the striatum of PD model mice, and the motor behavior symptoms of the mice were improved [38]. Another team achieved significant behavioral improvements by knocking down the PTBP1 gene in astrocytes in the striatum/nigrostriatal-dense part of PD model mice, resulting in dopaminergic neurons [44]. In a mouse spinal cord injury model, researchers promoted astrocyte transdifferentiation into neurons by lentiviral expression of Sox2+BDNF+NOG with concomitant knockdown of P53 expression, and approximately 5000 NeuN+ neurons were born around the injury, 80% of which were excitatory neurons [17]. Later, another researcher transformed NG2 glial cells in the spinal cord into neurons by expressing Sox2/P75-2 through a lentiviral system. They found a reduction in both volume and surface area of glial scars in the mouse model and improved behavior after 14 weeks [48].

## 6. Evidence of the Origin of Newborn Neurons

Whether the origin of the newborn neurons is astrocyte derived or endogenous neurogenesis is a question that researchers need to firstly demonstrate in this field. Most experiments now use the tamoxifen-induced Aldh1l1-CreERT2; R26R-YFP strain of mice and the mGfap-Cre; R26R-YFP line, which is specifically labeled for striatal astrocytes [15,17,26,28,40]. However, it has been shown that the cellular origin of NeuroD1-induced neurons is endogenous and that sequential BrdU labeling after brain injury fails to deliver the contribution of reactive glial cells to mCherry+ neurons [49].

## 7. Outlook

In summary, the induction of astrocyte transdifferentiation into neurons using transcription factors, miRNAs, or small molecule compounds in vivo or in vitro has gained some achievements. However, many gaps need to be bridged before there can be effective clinical translation such as improving the transdifferentiation efficiency, migrating, and homing the transdifferentiated neurons to the ischemic lesion, and limitating the tumorigenicity of the induced generated cells. Whether multiple injections or multiple doses have a positive effect on the prognosis of stroke should also be explored. Moreover, the introduction of transcription factors and miRNAs mostly requires viruses as vectors, which poses a challenge to the safety of this method. Although adenovirus shows good safety as a vector, its transfection efficiency is low and requires a high viral titer. In contrast, small-molecule, compound-induced reprogramming has attracted widespread attention because of its ease of synthesis, preservation, lower cost, and increased safety. However, the difficulty of using them remains high due to their diversity and complexity, so more investigations are needed to improve efficiency and reduce induced mutations. Moreover, all studies have been conducted in vitro or with mouse neural tissue, and none have been demonstrated in clinical practice. However, it is undeniable that the transdifferentiation of adult cells into neurons provides a new therapeutic idea for the treatment of ischemic stroke. If these problems can be solved, this treatment modality will significantly benefit patients.

## Figures and Tables

**Table 1:** Astrocyte transdifferentiation.

In Vivo/In Vitro	Original Cell	Target Cell	Reprogramming Factor	Efficiency	Ref
in vitro	Mouse Ast	Neurons	VCR	NeuN+ > 20%	[21]
in vitro	Mouse Ast	Neurons	Ngn2, Mash1	Ngn2 > 70% Mash1 > 30%	[24]
in vivo	Mouse Ast	GABAergic neurons Glutamate neurons	Neurog2/Dlx2	Ngn2 > 70% Dlx2 > 35.9%	[26]
in vivo	Mouse Ast	Glutamate neurons	NeuroD1	7-day conversion efficiency: >90%	[27]
in vitro	Mouse Ast	GABAergic neurons	Ascl1	13.2 ± 4.2% glutamatergic neurons 6.5 ± 2.2% GABAergic neurons	[28]
in vivo	Mouse Ast	GABAergic neurons Glutamate neurons	NeuroD1	17-day conversion efficiency: >70%	[29]
in vitro	Mouse Ast	Neurons	NeuroD1	66%	[30]
in vivo	Mouse Ast	Neurons	NeuroD1	2.42%	[31]
in vivo	Rhesus astrocytes	Neurons	NeuroD1	42-day conversion efficiency: >90%	[32]
in vitro	Human Ast/Mouse Ast	Dopaminergic neurons	NeuroD1 + Ascl1 + Lmx1a + miR128	16%	[38]
in vitro	Adult Ast	Cholinergic neuron Glutamate neurons	Small molecule compounds	8%	[40]
in vitro	Human Ast	Neurons	Small molecule compounds	68.7 ± 4.2%	[41]
in vitro	Human fetal astrocytes	Glutamate neurons GABAergic neurons	Small molecule compounds	40%	[42]
in vivo	Mouse Ast	Glutamate neurons GABAergic neurons	Small molecule compounds	Striatum: 11.3% Cerebral cortex: 10.7%	[43]
in vivo	Mouse Ast	Dopaminergic neurons	AAV2 system downgrades PTBP1	80% NeuN+ neurons, 30%~35% TH+ neurons	[44]

Ast: Astrocyte.

## Data Availability

Not applicable.
